# OCT4 increases BIRC5 and CCND1 expression and promotes cancer progression in hepatocellular carcinoma

**DOI:** 10.1186/1471-2407-13-82

**Published:** 2013-02-22

**Authors:** Lu Cao, Chunguang Li, Shuwen Shen, Yan Yan, Weidan Ji, Jinghan Wang, Haihua Qian, Xiaoqing Jiang, Zhigang Li, Mengchao Wu, Ying Zhang, Changqing Su

**Affiliations:** 1 Department of Molecular Oncology, Eastern Hepatobiliary Surgical Hospital & Institute, Second Military Medical University, 200438, Shanghai, China; 2 Department of Thoracic Surgery, Changhai Hospital, Second Military Medical University, 200438, Shanghai, China; 3 Department of Pathology, Cancer Center of PLA, Nanjing 81 Hospital, 210002, Nanjing, China

**Keywords:** Transcription factor, Cell cycle, Cell apoptosis, Cancer biotherapy, Hepatocellular carcinoma

## Abstract

**Background:**

OCT4 and BIRC5 are preferentially expressed in human cancer cells and mediate cancer cell survival and tumor maintenance. However, the molecular mechanism that regulates OCT4 and BIRC5 expression is not well characterized.

**Methods:**

By manipulating OCT4 and BIRC5 expression in hepatocellular carcinoma (HCC) cell lines, the regulatory mechanism of OCT4 on BIRC5 and CCND1 were investigated.

**Results:**

Increasing or decreasing OCT4 expression could enhance or suppress BIRC5 expression, respectively, by regulating the activity of BIRC5 promoter. Because there is no binding site for OCT4 within BIRC5 promoter, the effect of OCT4 on BIRC5 promoter is indirect. An octamer motif for OCT4 in the CCND1 promoter has directly and partly participated in the regulation of CCND1 promoter activity, suggesting that OCT4 also could upregulated the expression of CCND1. Co-suppression of OCT4 and BIRC5 induced cancer cell apoptosis and cell cycle arrest, thereby efficiently inhibiting the proliferative activity of cancer cells and suppressing the growth of HCC xenogrfts in nude mice.

**Conclusion:**

OCT4 can upregulate BIRC5 and CCND1 expression by increasing their promoter activity. These factors collusively promotes HCC cell proliferation, and co-suppression of OCT4 and BIRC5 is potentially beneficial for HCC treatment.

## Background

Recurrence and metastasis of hepatocellular carcinoma (HCC) depend on the persistent proliferative activity of cancer cells. BIRC5, also called Survivin, has been shown to play a pivotal role in cancers by influencing cell division and proliferation and by inhibiting apoptosis [1]. Many studies using clinical specimens have shown that BIRC5 is invariably overexpressed in a majority of human cancers and is linked to poor patient prognosis but is rarely expressed in normal tissues [2]. Based on the abnormally high activation of BIRC5 during carcinogenesis in various types of cancers, treatment that targets BIRC5 has been increasingly recognized as a promising therapy for various cancers. However, when the anti-BIRC5 agent is used alone, the long-term efficacy remains uncertain and is variable for different types of cancers; tumors have always relapsed and regrown in later stages after treatment. Many factors are involved in the regulation of BIRC5 expression and function, and all of these factors influence the efficacy of BIRC5-targeting strategies. We have found that P16 reactivation in HCC cells down-regulates BIRC5 expression and limits CDK4 import into nuclei, and then exhibits the effect of cell cycle arrest and the induction of detachment-induced apoptosis [3]. Another research group has reported that the octamer-binding transcription factor 4 (OCT4) regulates BIRC5 expression, which was dramatically decreased in OCT4 knockdown murine embryonic stem cells [4].

OCT4, a member of the POU-domain transcription factor family, plays a pivotal role in the regulation and maintenance of the cellular pluripotent state [5,6]. More recently, the expression of OCT4 in human cancer cells has been demonstrated [7-9]. OCT4 activates the transcription of downstream target genes via its octamer motif (5^′^-ATGCAAAT-3^′^) [10], and various genes have been reported to have the OCT4 binding sites, including fibroblast growth factor 4 (FGF-4) [11]. Both global chromatin immunoprecipitation assays and global expression profiling have been used to characterize the gene regulatory network governed by OCT4, and a large list of candidate target genes, whose regulatory sequences are recognized by OCT4, has been generated [12,13]. OCT4 also can bind to other similar sequence motifs [13,14]. However, many downstream target genes do not have OCT4 motifs but might still be valid candidates as putative indirect targets of OCT4. The BIRC5 promoter was reported to not have binding sites for OCT4, although OCT4 knockdown in murine embryonic stem cells has been shown to decrease the expression level of BIRC5 protein, suggesting an indirect effect of OCT4 on BIRC5 expression [4].

In this study, we investigated the regulatory mechanism and significance of OCT4 on BIRC5 and CCND1 expression in HCC. Although the roles for BIRC5 and OCT4 in cancers are well-recognized in a number of previous studies, we gave the first evidence that OCT4 indirectly manipulates the expression and function of BIRC5, and also directly upregulates the expression of CCND1. These factors collude to promote cancer cell proliferation and resist cancer cell apoptosis. This innovative finding provides new insight into the regulation of OCT4 on CCND1 expression through a previously unidentified mechanism and indicates a variety of novel biological and prognostic markers, as well as potential therapeutic targets, for cancer diagnosis and treatment.

## Methods

### Vectors and adenoviruses

Vectors expressing the specific small hairpin RNA (shRNA), including BIRC5-shRNA, OCT4-shRNA and Dual-shRNA, were synthesized by and purchased from Wuhan Genesil Biotechnology Co., Ltd. (Wuhan, China). The 19-nt sense DNA of BIRC5-shRNA (5^′^-GAAAGTGCGCCGTGCCATC-3^′^) targets base pairs 436–454 of the BIRC5 gene (HSU75285), and the 19-nt sense DNA of OCT4-shRNA (5^′^-CCCTCACTTCACTGCACTG-3^′^) targets base pairs 1233–1253 of the OCT4 gene (DQ486513.1). Both gene elements were controlled by the U6 promoter. A mock control shRNA vector (Ctrl-shRNA, 5^′^-GACTTCATAAGGCGCATGC-3^′^) was concomitantly constructed.

Full-length OCT4 cDNA was cloned into pDC315 (Microbix Biosystems, Ontario, Canada) at the *EcoR*I and *Sal*I sites to generate pDC315-OCT4. Sequences of the shRNA loop were digested from shRNA vectors and then inserted into pDC315 at the *Bam*HI and *Sal*I sites to obtain pDC315-shBIRC5, pDC315-shOCT4 and pDual-shRNA. The plasmids pDC315-OCT4, pDC315-shBIRC5, pDC315-shOCT4 and pDual-shRNA were transfected into HEK293 cells (Microbix Biosystems, Ontario, Canada) using the Lipofectamine 2000 reagent (Invitrogen Corporation Shanghai Representative Office, Shanghai, China) together with the type 5 adenovirus packaging plasmid pBHGloxdelE13cre (Microbix Biosystems, Ontario, Canada) to generate a set of adenoviruses named Ad5-OCT4, Ad5-shBIRC5, Ad5-shOCT4 and AdDual-shRNA.

### Promoter-regulated reporter gene vectors

The luciferase plasmid pSRVN-Luc, in which luciferase expression was under the control of the BIRC5 promoter (nucleotides 1824–2800, GenBank U75285), was kindly provided by Himanshu Garg (Center of Excellence for Infectious Disease, Texas Tech University Health Sciences Center, TX). The BIRC5 promoter was amplified with the indicated primers (P1: 5^′^-cgGCTAGCcatagaaccagag-3^′^; P2: 5^′^-gaAGATCTgccgccgccgccacct-3^′^) and inserted into an EGFP plasmid at the *Nhe*I and *Bgl*II sites to yield pSRVN-EGFP.

The wild type CCND1 promoter (wPro; nucleotides 2501–3178, GenBank Z29078.1) was amplified from HepG2 genomic DNA with the indicated primers (P3: 5^′^-cgGGATCCagattctttggccgtctgtc-3^′^; P4: 5^′^-cgGAATTCAAGCTTggctggggctcttc ctg-3^′^) and then inserted into the luciferase plasmid at the *Bgl*II and *Hind*III sites to generate pGL3wPro-Luc. The base pairs “ATTTGCAT” in wPro from −252 to −245 were replaced by base pairs “ATCTGTAT” and “ATTTGAAATGCAAAT (PORE motif)” to construct the motif-mutated promoter (mPro) and motif-enhanced promoter (ePro), respectively. The mPro-controlled luciferase plasmid (pGL3mPro-Luc) and the ePro-controlled luciferase plasmid (pGL3ePro-Luc) were generated.

### Animal experiments

Hep3B cells were subcutaneously injected into the right flanks of BALB/c (nu/nu) mice (10^7^ cells per mouse) (Shanghai Experimental Animal Center, Chinese Academy of Sciences, Shanghai, China) to establish xenograft tumors. Ten weeks later, mice were separated randomly into 4 groups (Dual-shRNA, BIRC5-shRNA, OCT4-shRNA and blank control groups) with 5 mice per group. Mice in the virus-treated groups were given 5 viral intratumoral injections once every other day for a total dosage of 10^9^ pfu virus per mouse. Mice in the control group were given the same volume of viral preservation solution (10 mmol/L Tris–HCl pH 8.0, 2 mmol/L MgCl_2_, 4% sucrose). Tumor size was measured regularly, and the tumor volume was estimated with the formula “*a* × *b*^2^ × 0.5”, in which *a* and *b* represent the maximal and minimal diameters, respectively. The animal welfare guidelines for the care and use of laboratory animals were approved by the Animal Care Committee of Second Military Medical University (No. SCXK2009-0003).

### Statistical analysis

The experimental data were statistically analyzed using the student's *t* test, and http://two-way analysis of variance (ANOVA) according to the properties of the data. All tests were performed using the PASW Statistics 18.0 software. *P* < 0.05 was considered statistically significant. The detailed methods is available at Journal’s website as Additional file 1: Supplementary Methods.

## Results

### BIRC5 expression was associated with OCT4 in HCC cells

**Figure 1:**
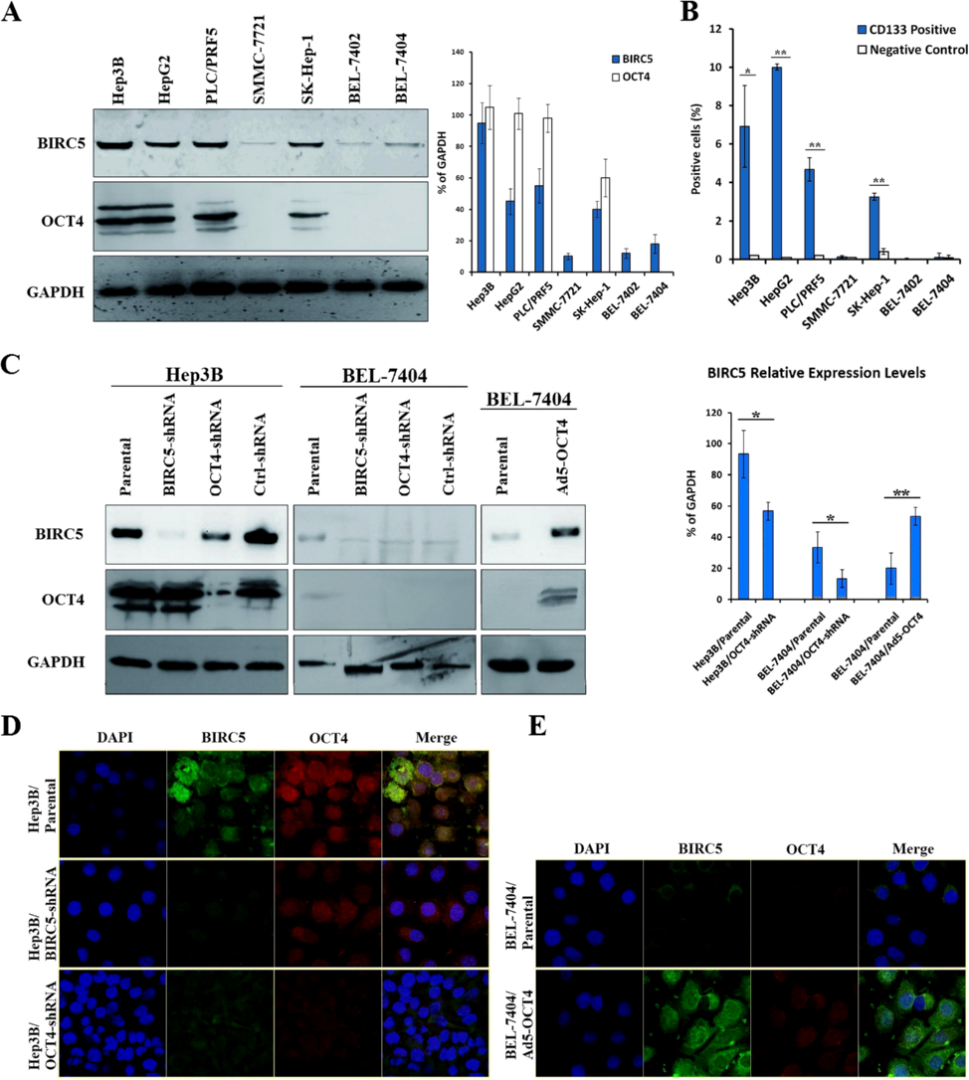
**BIRC5 and OCT4 expression in HCC cell lines.** (**A**) Cell lines were cultured in 6-well plates at a density of 10^5^ cells/well for 48 h, and then harvested for measuring expression of BIRC5 and OCT4 by Western blotting. Glyceraldehyde-3-phosphate dehydrogenase (GAPDH) was used as the loading control, and densitometry analysis was performed to show BIRC5 and OCT4 expression levels normalized with GAPDH density. (**B**) Cells were harvested, the CD133-positive cells were quantified by flow cytometry and showed in percentages of total cells counted; *, *P <* 0.05; **, *P <* 0.01. (**C)** Cells were infected with Ad5-OCT4 at an MOI of 20 pfu/cell, or transfected with shRNA vectors at 20 μg/well. BIRC5 and OCT4 expression was measured by Western blotting. Densitometry analysis was performed to show BIRC5 expression levels, normalized with GAPDH density; *, *P <* 0.05; **, *P <* 0.01. (**D, E)** The parental, adenovirus-infected and shRNA-transfected HCC cells, including Hep3B (**D**) and BEL-7404 (**E**), were cultured in Lab-Tek chamber at a density of 10^4^ cells/well for 48 h, fixed in 4% formaldehyde for 30 min, and labeled by the indicated fluorescent antibodies. DAPI was used to stain cellular nuclei, original magnification 400 × .

To verify the regulation of OCT4 and BIRC5 expression in HCC cells, a plasmid vector expressing BIRC5-specific small hairpin RNA (BIRC5-shRNA) or OCT4-specific small hairpin RNA (OCT4-shRNA) and an adenovirus vector expressing OCT4 (Ad5-OCT4) were constructed and used to manipulate the expression of BIRC5 and OCT4 in HCC cells. Based on Western blotting results, the parental HCC cells, including Hep3B, HepG2, PLC/PRF5, SMMC-7721, BEL-7402, SK-Hep-1 and BEL-7404, were all positive for BIRC5, although the expression levels were lower in SMMC-7721, BEL-7402 and BEL-7404 cells than in Hep3B, HepG2, PLC/PRF5 and SK-Hep-1 cells. Regarding OCT4 expression, Hep3B, HepG2, PLC/PRF5 and SK-Hep-1 cells were positive and BEL-7402 and BEL-7404 cells were negative (Figure 1A). Using flow cytometric analysis, we analyzed CD133 expression of HCC cell lines. The results showed that the percentages of CD133-positive cells were http://consistent with OCT4 expression in these cell lines (Figure 1B).

Because Hep3B cells expressed both BIRC5 and OCT4, and BEL-7404 cells expressed BIRC5 but not OCT4, both cell lines were transfected with BIRC5-shRNA and OCT4-shRNA vectors, and BEL-7404 cells were infected with Ad5-OCT4. After transfection with BIRC5-shRNA, BIRC5 expression was considerably down-regulated in Hep3B and BEL-7404 cells, whereas OCT4 expression was not affected. However, after transfection with OCT4-shRNA, OCT4 expression was inhibited; BIRC5 was also clearly down-regulated even in the OCT4-negative BEL-7404 cells. To further validate this observation, BEL-7404 cells were infected with Ad5-OCT4, and the results demonstrated that adenovirus-mediated overexpression of OCT4 http://leads http://to a significant up-regulation of BIRC5 (Figure 1C).

The dynamic localization and expression levels of BIRC5 and OCT4 were analyzed in HCC cells by immunofluorescent labeling. BIRC5 expression was observed in the cytoplasm of Hep3B parental cells and was inhibited after transfection with BIRC5-shRNA. OCT4 expression was detected in the nuclei of Hep3B parental cells and was inhibited after transfection with OCT4-shRNA. OCT4-shRNA transfection consequently resulted in the down-regulation of BIRC5 and also induced cell apoptosis, as indicated by the occurrence of nuclear condensation and apoptotic bodies (Figure 1D). In BEL-7404 cells, BIRC5 expression was increased by adenovirus-mediated OCT4 expression in the cytoplasm and nuclei (Figure 1E).

### OCT4 upregulated BIRC5 expression by activating the BIRC5 promoter

**Figure 2:**
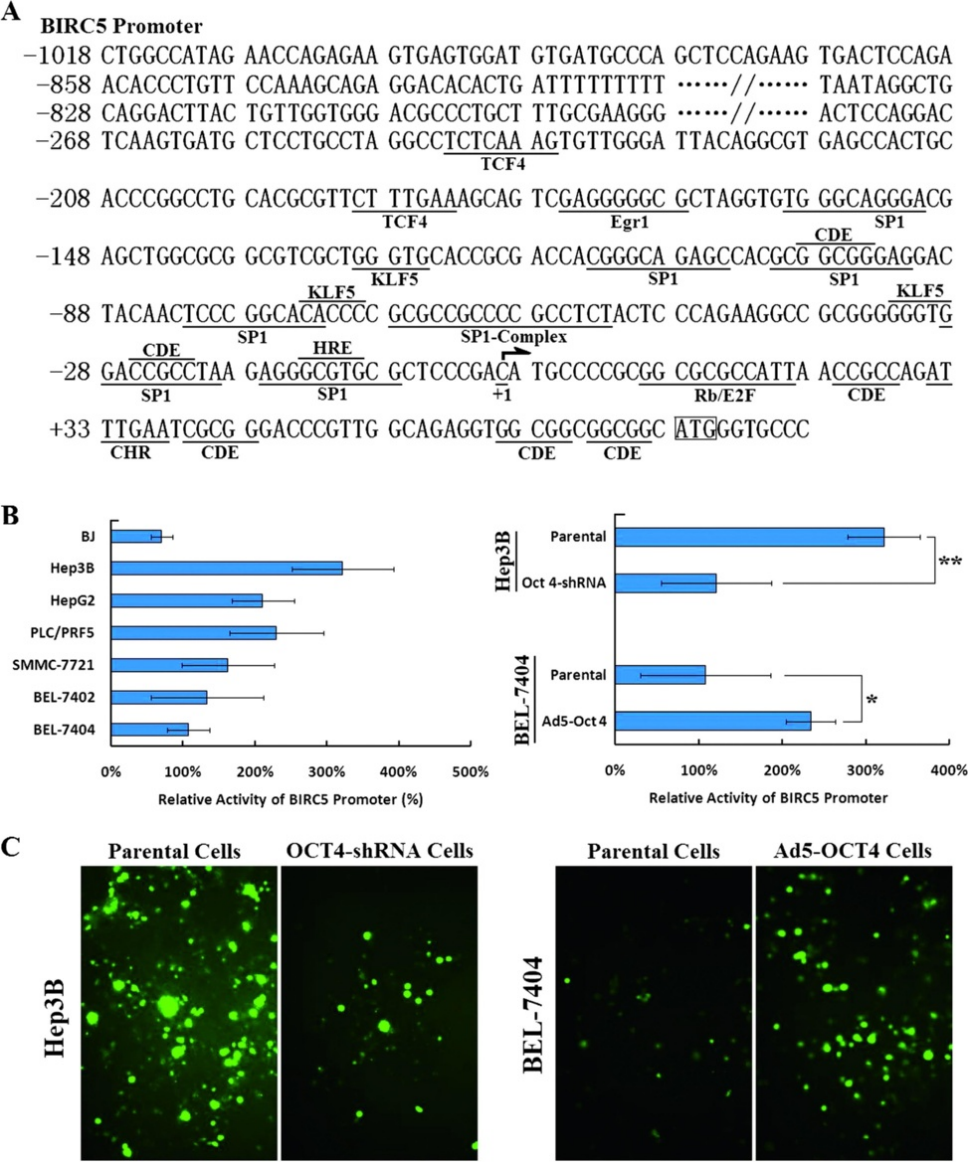
**BIRC5 promoter activity in HCC cells.** (**A**) Scheme of binding sites for transcription factors in the BIRC5 proximal promoter region. (**B**) Cell lines, including the indicated parental, adenovirus-infected and shRNA-transfected cells, were seeded on 24-well plates at a density of 5 × 10^4^ cells/well and transfected with the BIRC5 promoter-driven luciferase plasmid pSRVN-Luc (200 ng/well). The relative activity of BIRC5 promoter in HCC cells was measured and shown in histograms; *, *P <* 0.05; **, *P <* 0.01. (**C)** Cells were seeded on 96-well plates at a density of 5 × 10^3^ cells/well, transfected with the BIRC5 promoter-driven EGFP plasmid pSRVN-EGFP (2 μg/well), and observed under a fluorescent microscope 48 h later, original magnification 200 × .

The expression of BIRC5 varied with changes in the expression of OCT4, suggesting that BIRC5 is under the control of OCT4. To explore the superior-subordinate relationship between BIRC5 and OCT4, the BIRC5 proximal promoter was amplified (Figure 2A) and cloned into luciferase reporter gene and enhanced green fluorescent protein (EGFP) gene vectors, and the BIRC5 promoter activity was then measured in HCC cells. Compared to the normal BJ cell line, the relative activity of BIRC5 promoter in HCC cells was higher, particularly in the Hep3B, PLC/PRF5 and HepG2 HCC cell lines. OCT4-negative cells, such as BEL-7402 and BEL-7404, also presented high BIRC5 promoter activity compared to BJ cells (Figure 2B). After the inhibition of OCT4 expression with OCT4-shRNA, the relative BIRC5 promoter activity in Hep3B cells was significantly suppressed (*p =* 0.0099). Adenovirus-induced OCT4 expression in BEL-7404 cells resulted in an increase in BIRC5 promoter activity (*P =* 0.0199). Results using the EGFP vector were consistent with those using the luciferase vector (Figure 2C).

### OCT4 upregulated CCND1 expression by activating the CCND1 promoter

**Figure 3:**
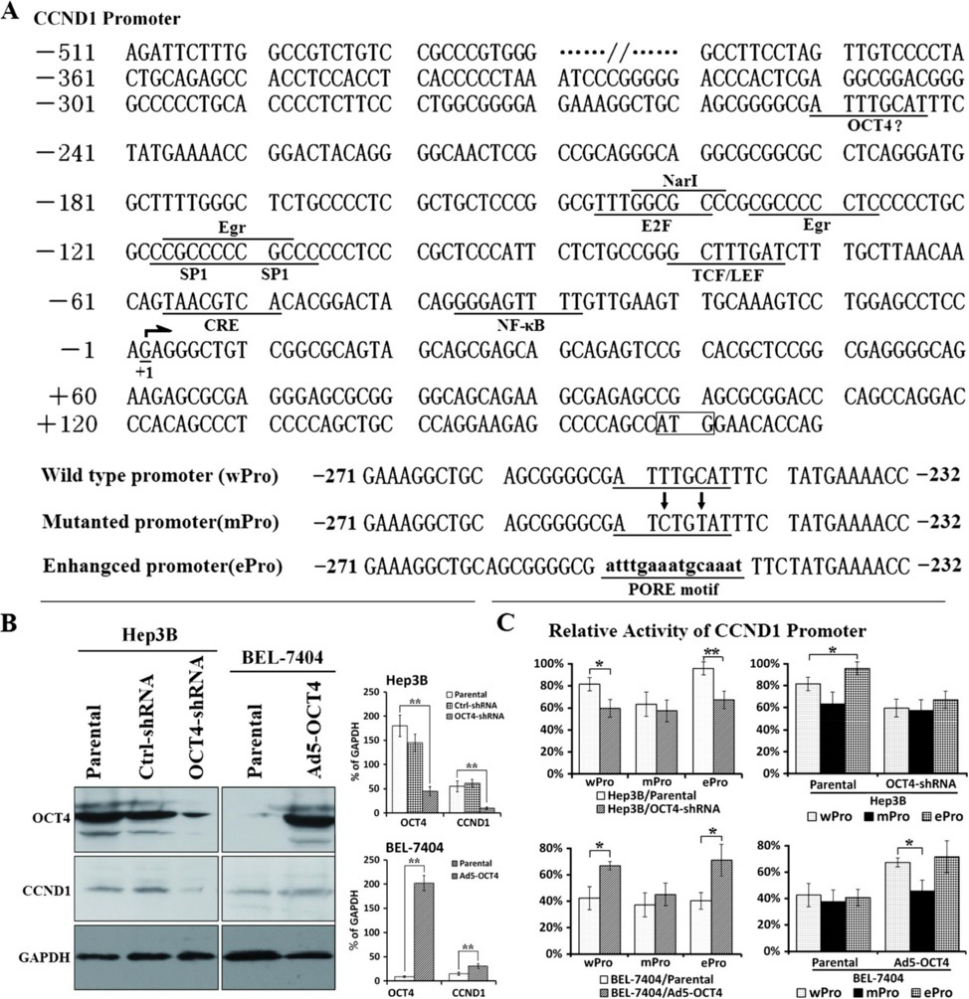
**CCND1 promoter activity in HCC cells.** (**A**) The wild type CCND1 promoter (wPro) containing the transcription factor binding sites was cloned from HepG2 DNA genome. From the wPro, the motif-mutated promoter (mPro) and motif-enhanced promoter (ePro) within −252 to −245 were amplified and used to construct the CCND1 promoter-driven luciferase plasmids, pGL3wPro-Luc, pGL3mPro-Luc and pGL3ePro-Luc. (**B**) CCND1 and OCT4 expression was detected by Western blotting in HCC cells. GAPDH was used as the loading control, and densitometry analysis was performed to show CCND1 and OCT4 expression levels normalized with GAPDH density; **, *P <* 0.01. (**C**) The indicated parental, adenovirus-infected and shRNA-transfected cells were seeded on 24-well plates at a density of 5 × 10^4^ cells/well and transfected with the plasmids pGL3wPro-Luc, pGL3mPro-Luc and pGL3ePro-Luc at 200 ng/well. The relative activity of CCND1 promoter in HCC cells was measured and shown in histograms; *, *P <* 0.05; **, *P <* 0.01.

OCT4 can control cell cycle by up-regulating target genes associated with cell cycle [10]. Therefore, we screened for OCT4 binding sites in the promoter regions of these cell cycle regulators and found that the CCND1 proximal promoter contains an octamer motif at −252 to −245 (Figure 3A). After CCND1 expression in HCC cells was confirmed by Western blotting, we cloned the wild type, motif-mutated, and motif-enhanced CCND1 promoters and investigated OCT4 regulatory function using the CCND1 promoter.

Both Hep3B and BEL-7404 cells were positive for CCND1 expression. Silencing of OCT4 expression by OCT4-shRNA resulted in the down-regulation of CCND1 expression in Hep3B cells, whereas enhanced OCT4 expression by Ad5-OCT4 infection led to an up-regulation of CCND1 expression in BEL-7404 cells (Figure 3B). The relative CCND1 promoter activity was detected using the luciferase reporter assay (Figure 3C). In OCT4-positive Hep3B cells, the activity of mutated promoter (mPro) was slightly lower compared to that of the wild type promoter (wPro) (*P =* 0.0644), whereas the activity of the enhanced promoter (ePro) with a PORE motif was significantly higher (*P =* 0.0446). After the silencing of OCT4 expression, wPro or ePro activity was decreased relative to the parental cells (wPro: *P =* 0.0192; ePro: *P =* 0.0075). In OCT4-negative BEL-7404 cells, the three promoters consistently maintained a similar level of activity. However, when OCT4 was re-expressed, wPro and ePro activity was significantly increased (wPro: *P =* 0.0106; ePro: *P =* 0.0167, compared with the parental cells).

### Co-suppression of OCT4 and BIRC5 inhibits Hep3B cell growth and induces cell apoptosis

**Figure 4:**
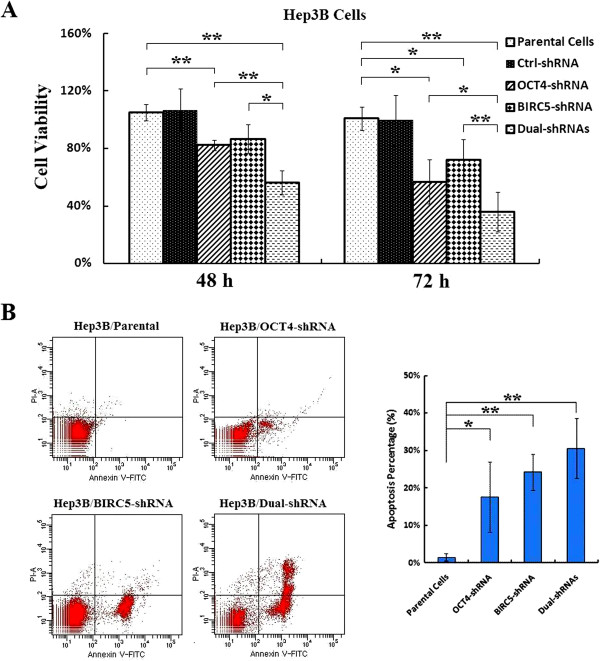
**Cell growth inhibition and cell apoptosis induced by co-suppression of OCT4 and BIRC5 in HCC cells.** (**A**) The parental and indicated shRNA-transfected Hep3B cells were cultured in 96-well plates at a density of 5 × 10^3^ cells/well for 48 and 72 h. Cell viability was measured by MTT assay at a wavelength of 570 nm with a reference wavelength of 655 nm and shown in histograms; *, *P <* 0.05; **, *P <* 0.01. (**B**) The parental, adenovirus-infected and shRNA-transfected Hep3B cells at 10^6^ cells/ml were stained with PI and Annexin V-FITC for detection of cell apoptosis. Percentages of cell apoptosis were shown in histograms; *, *P <* 0.05; **, *P <* 0.01.

To further explore the functions of OCT4 and BIRC5 in cancer cell proliferation, a dual-target shRNA vector (Dual-shRNA) that targeted OCT4 and BIRC5 was constructed, and its inhibitory effect on HCC cells was compared to that of the mono-target shRNA vectors. After transfection with the shRNA vectors, cancer cell viability was slightly lower with each of the vectors (Figure 4A). The inhibitory effect of OCT4-shRNA on Hep3B cell viability was stronger than that of BIRC5-shRNA, and the inhibitory effect of the Dual-shRNA was the strongest among the shRNA vectors tested. By Annexin V-FITC/PI labeling, the percentage of apoptotic cells (both Annexin V-FITC-positive/PI-negative and Annexin V-FITC-positive/PI-positive) in the Dual-shRNA-transfected population was shown higher than the percentage of the other two shRNA vector-transfected cell populations (Figure 4B).

### Co-suppression of OCT4 and BIRC5 induces Hep3B cell cycle arrest

**Figure 5:**
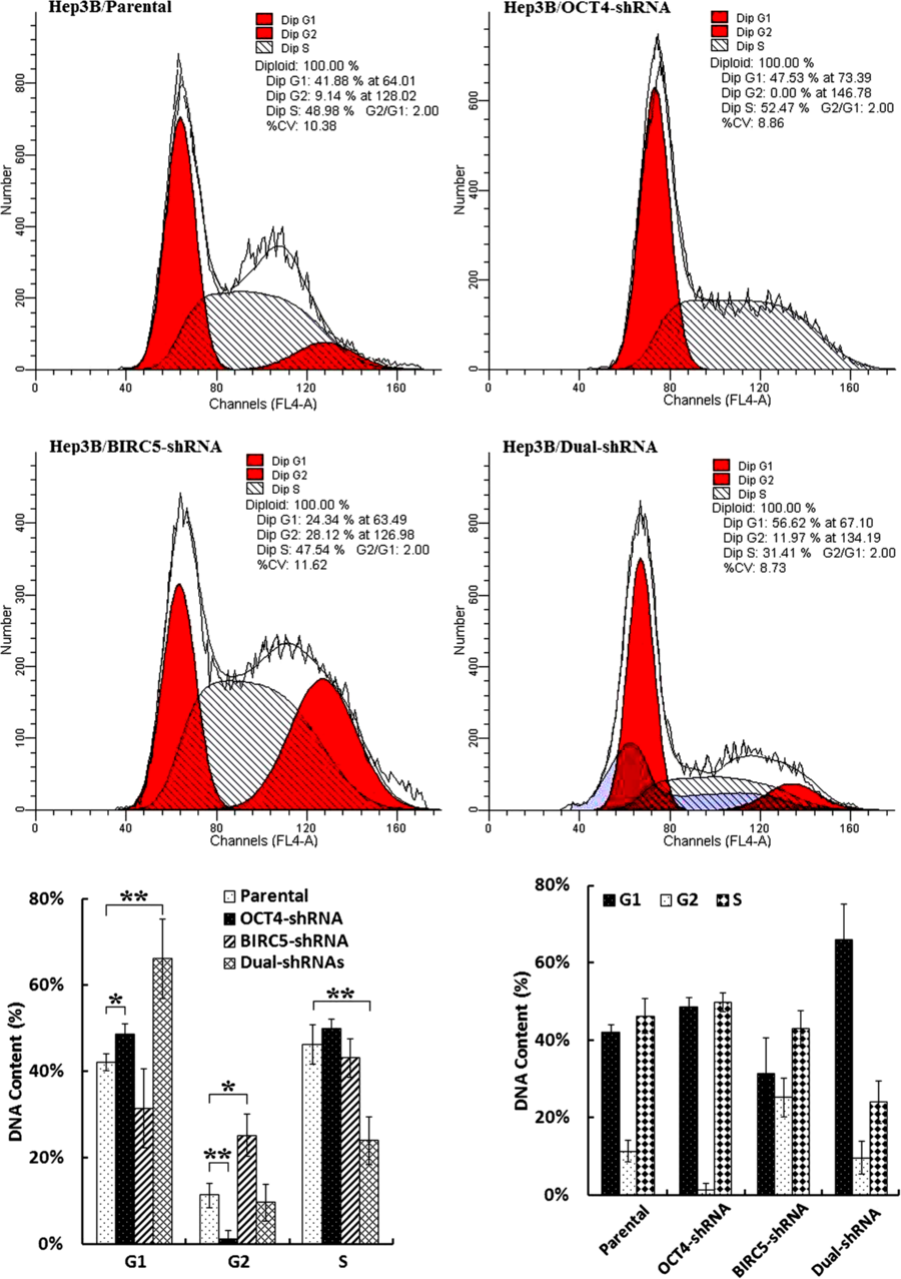
**Cell cycle arrest induced by co-suppression of OCT4 and BIRC5 in HCC cells.** The parental, adenovirus-infected and shRNA-transfected Hep3B cells at 10^6^ cells/ml were stained with PI, and analyzed by flow cytometry. Data of cell cycle were shown in histograms; *, *P <* 0.05; **, *P <* 0.01.

The parental and shRNA-transfected Hep3B cells were then examined by flow cytometry using propidium iodide (PI) staining to identify phases of cell cycle arrest. Compared with the parental cells, OCT4-shRNA transfection resulted in a G1-phase arrest (*P* = 0.0193) and G2-phase decrease (*P* = 0.0067); BIRC5-shRNA transfection resulted in a G2-phase arrest (*P* = 0.0129), and Dual-shRNA transfection resulted in a distinct G1-phase arrest (*P* = 0.0072) and S-phase decrease (*P* = 0.0024; Figure 5).

### Simultaneously silencing of OCT4 and BIRC5 exhibits strong antitumor potency against HCC xenograft tumors in nude mice

**Figure 6:**
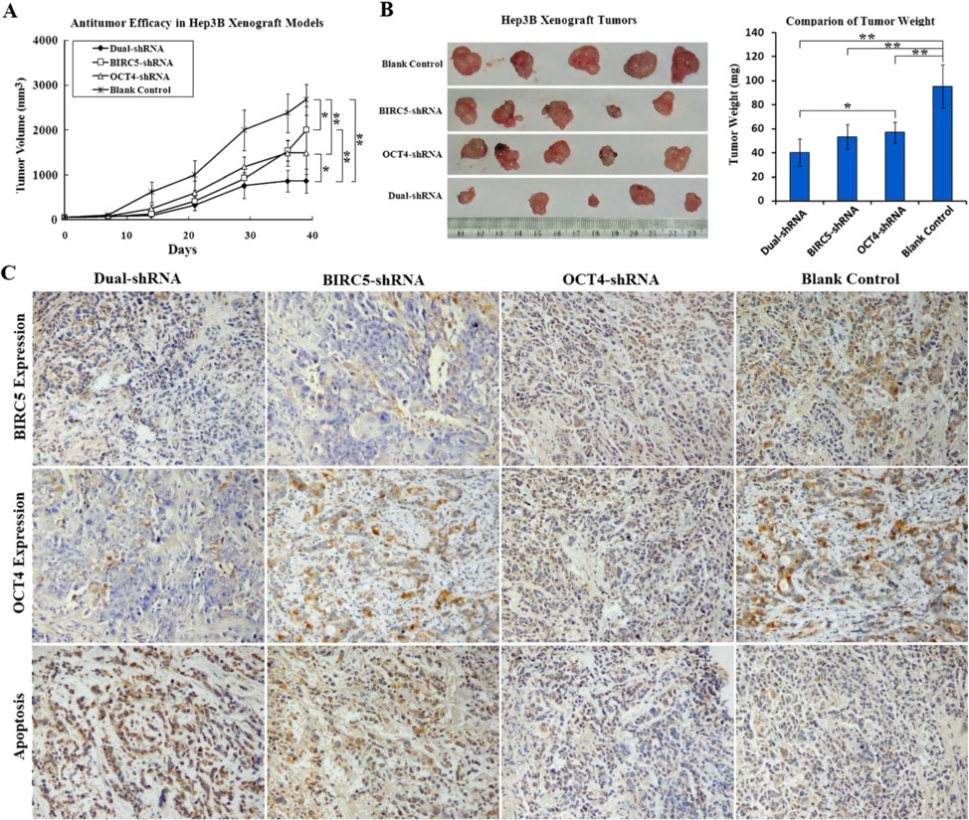
**Antitumour efficacy of OCT4 and BIRC5 co-suppression in mouse xenograft models. (A)** Hep3B cells were injected into BALB/c (nu/nu) mice for establishing xenograft tumours. Adenoviruses carrying Dual-shRNA, BIRC5-shRNA, OCT4-shRNA were used to treat tumours by intratumoural injections with a total dosage of 10^9^ pfu viruses per mouse, and the tumour volume was measured every week. (**B**) Observation was ended (day 39 after treatment) when the tumours were over 2,500 mm^3^. Mice were killed, tumours were removed and weighed; *, *P* < 0.05, n = 5; **, *P* < 0.01, n = 5. (**C**) The paraffin-embedded consecutive sections of tumours were detected for OCT4 and BIRC5 expression by immunohistochemistry and for counting apoptotic cells by TUNEL, original magnification 200 × .

Hep3B xenograft tumors were established in nude mice and infected with adenoviruses carrying OCT4-shRNA or/and BIRC5-shRNA. Compared with the control group, adenovirus with Dual-shRNA exerted the strongest inhibitory effect on tumor growth because the tumor inhibitory rate was 67.59% (*P =* 0.0001) compared to 44.54% (*P =* 0.0020) and 25.25% (*P =* 0.0418) in the OCT4-shRNA and BIRC5-shRNA groups, respectively (Figure 6A). After completing the treatments, tumors in the BIRC5-shRNA group gradually regrew, but those in the OCT4-shRNA group showed a longer-lasting growth-inhibitory effect. Adenovirus with Dual-shRNA exhibited the greatest antitumor potency (*P =* 0.0358 for the OCT4-shRNA group, *P =* 0.0025 for the BIRC5-shRNA group, versus the Dual-shRNA group). After the observation period, the mice were killed, and the tumors were removed. The tumor weights also indicated that the Dual-shRNA group had the greatest antitumor efficacy (Figure 6B).

Paraffin-embedded tumor sections were examined by immunohistochemistry and terminal deoxynucleotidyl transferase-mediated dUTP nick end labelling (TUNEL). In the blank control group, cancer cells were positive for OCT4 and BIRC5 expression, and few tumor cells were positive for TUNEL staining. However, in the BIRC5-shRNA group, BIRC5 expression was down-regulated and the apoptotic cell percentage was increased. When OCT4 expression was down-regulated by OCT4-shRNA in tumor cells, BIRC5 expression was also suppressed accompanied by an increase in the percentage of apoptotic cells. The Dual-shRNA simultaneously silenced OCT4 and BIRC5 expression and resulted in a significant increase in apoptotic cells in Hep3B xenograft tumors (Figure 6C).

## Discussion

As a member of the inhibitors of apoptosis protein (IAPs) family, BIRC5 is preferentially expressed in human cancer cells and has multiple functions, including the inhibition of cell apoptosis [1], control of the cell cycle [15,16], promotion of tumor angiogenesis [17,18], resistance to chemotherapy or radiotherapy [19], acceleration of metastasis and recurrence [20,21], and regulation of cancer cell autophagy [22], all of which favour cancer cell survival and tumor maintenance. Therefore, multiple strategies have been employed to target BIRC5 for cancer therapy by silencing BIRC5 expression with small interfering RNA [23] or antisense oligonucleotides [24], inhibiting the BIRC5 promoter activity with small-molecule antagonists [25], and interfering BIRC5 function with dominant-negative mutant forms of the protein [26]. Some of these strategies are being applied in clinical trials at various phases, and the initial results are promising when combined with other treatments, such as chemotherapy or radiotherapy [16,27]. Although certain strategies for cancer therapy targeting BIRC5 have shown a varied extent of antitumor efficacy, the potential benefit of single anti-BIRC5 treatment in different types of cancers is uncertain. Although the down-regulation of BIRC5 expression by anti-BIRC5 agents can inhibit the growth of cancer, tumors consistently obtain growth capabilities in later stages, demonstrating that this treatment approach remains poorly characterized and requires further study.

BIRC5 expression is precisely regulated at transcriptional and post-translational levels. The signal transducer and activator of transcription 3 (Stat-3), β-catenin-activated T-cell factor (TCF) transcription factor, hypoxia-inducible factor-1 alpha (HIF-1_α_) and Sp1 transcription factor promote BIRC5 expression by increasing BIRC5 promoter activity [28-31]. Sp1-mediated BIRC5 expression can be suppressed by p53 [32]. The stability of BIRC5 protein represents another potential method of regulating BIRC5 function. BIRC5 protein is phosphorylated at Thr34 by cdc2 kinase, which prevents BIRC5 proteosome-mediated clearance or degradation [33]. Recently, OCT4 was reported to have a regulatory effect on BIRC5 expression [4].

OCT4 belongs to the family of POU-domain transcription factors, which are involved in the regulation of cell growth and differentiation in a variety of tissues [11,34]. Many studies have shown that OCT4 expression is restricted to germline and pregastrulation embryos and also to embryonal carcinomas and testicular germ cell tumors [7], but not expressed in mature somatic cells. Further evidence has shown that some cancer cells, such as breast, bladder, prostate, liver, head and neck squamous cell cancer and non-small cell lung cancers, are positive for OCT4 expression [7-9,35-39]. Therefore, OCT4 acts as a multifunctional factor not only in stem cells but also in many cancers, and the expression of OCT4 causes more malignant histological phenotypes, including rapid progression, great metastasis, and short cancer-related survival. However, one study unexpectedly found that adult human peripheral blood mononuclear cells, which are genetically stable and mainly terminally differentiated cells with a limited lifespan, express OCT4; this finding challenges the paradigm of OCT4 as a marker of pure stem cells and provides novel insight into the role of OCT4 in fully differentiated cells [11]. OCT4 functions by directly or indirectly activating a series of downstream target genes. By characterizing the genes in OCT4-mediated regulatory networks, it has been found that many candidate target genes that are directly regulated by OCT4 have an OCT4-binding octamer motif [10]. However, a large number of target genes, such as BIRC5 [4], have no OCT4 motifs and might be indirectly regulated by OCT4. Therefore, transcriptional regulation of target genes by OCT4 is very complicated, and it is necessary to understand the key gene network that maintains cell pluripotency in embryo development and governs cell differentiation and proliferation in cancer progression.

To clarify the relationship between OCT4 and BIRC5 in HCC, we first analyzed the OCT4 and BIRC5 expression levels in HCC cell lines, including Hep3B, HepG2, PLC/PRF5, SMMC-7721, BEL-7402 and BEL-7404. All cell lines were positive for BIRC5 expression, although only the Hep3B, HepG2 and PLC/PRF5 cells were positive for OCT4 expression; SMMC-7721 cells were weakly positive for OCT4 expression. OCT4 and BIRC5 expression was also investigated by immunohistochemistry in 49 pairs of cancer and liver tissues taken from HCC patients. They were overexpressed in HCC compared with the corresponding liver tissues (Additional file 2: Table S1). BIRC5 immunostaining was mainly localized in cancer cell cytoplasm and nuclei, and OCT4 expression was localized in cancer cell nuclei (Additional file 3: Figure S1).

We found that the expression levels of OCT4 in HCC cell lines were http://consistent with the percentages of CD133-positive cells, suggesting that OCT4 expression might be related to CD133 expression. By manipulating the expression of OCT4 and BIRC5, we found that BIRC5 expression silencing did not influence OCT4 expression in Hep3B and BEL-7404 cells. However, down-regulation of OCT4 expression inhibited BIRC5 expression, even in the OCT4-negative BEL-7404 cells, and increasing OCT4 expression by infection with adenovirus carrying the OCT4 gene in BEL-7404 cells up-regulated BIRC5 expression. In exploring the superior-subordinate relationship between BIRC5 and OCT4, we found that the relative activity of the BIRC5 promoter in HCC cells was controlled by OCT4. These results http://seemingly demonstrated that BIRC5 is a downstream target gene of OCT4.

Functional binding sites for the transcription factors SP1, KLF5, HIF-1α, Rb/E2F, TCF4 and Egr1 have been found in the BIRC5 gene promoter, suggesting that these factors regulate BIRC5 gene expression [40]. However, a binding site for OCT4 is not found in the BIRC5 promoter region, suggesting that OCT4 may indirectly regulate BIRC5 expression [13,40]. In addition to the Rb suppressor and E2F activators (i.e., E2F1, E2F2 and E2F3) that bind directly to the BIRC5 promoter and regulate BIRC5 transcription [41], the regulatory proteins CDK4, SKP2, Rad51, BRCA2, E2F-DP1, CCND1, Stat3, Rb and p21 can activate the SP1 promoter [42], which then indirectly leads to an increase in BIRC5 expression. These factors are all involved in cell cycle regulation. In addition, OCT4 modulates the cell cycle by up-regulating CDKN1B, CDKN1C, CDK6 and MAPK4 [10]. Coincidentally, by screening the binding sites in the promoter regions of these cell cycle regulators, we found an octamer motif for OCT4 at −252 to −245 in the CCND1 proximal promoter. Further studies have confirmed that CCND1 expression and promoter activity is strictly correlated with OCT4 expression levels in OCT4-positive Hep3B cells. When the OCT4 motif in the CCND1 promoter was mutated or modified with a PORE motif that could bind two OCT4 molecules, the promoter activity was suppressed or enhanced, respectively. We also observed high CCND1 promoter activity in OCT4-negative BEL-7404 cells. These results suggested that the OCT4 motif might participate in the regulation of CCND1 promoter activity, and that there are other factors that regulate CCND1 promoter activity in HCC cells.

In the *in vitro* experiments, silencing of BIRC5 expression effectively induced apoptosis and cell cycle arrest in HCC cells, thereby inhibiting cancer cell proliferation and decreasing cancer cell viability. Co-suppression of OCT4 and BIRC5 further enhanced the inhibitory effect on cancer cell proliferation. In the *in vivo* experiments, BIRC5-shRNA expression inhibited the growth of HCC xenograft tumors by inducing cell apoptosis, although tumor growth was restored in the late stage after the adenovirus injections ceased. The Dual-shRNA that targeted both OCT4 and BIRC5 inhibited tumor growth with great efficiency for a long period of time. These results showed that OCT4 and BIRC5 collusively educe cell proliferation. Clinical follow-up information also demonstrated that the HCC patients who showed co-expression of OCT4 and BIRC5 in cancer tissues had poorer disease-free survival (DFS) and overall survival (OS) than patients who were negative for both OCT4 and BIRC5 (Additional file 4: Figure S2).

## Conclusions

This study demonstrated that OCT4 is an upstream gene that indirectly upregulates BIRC5 expression, and directly upregulates CCND1 expression. These factors collude to promote the proliferation of cancer cells and contribute to the poor prognosis of HCC patients. The innovative finding provides new insight into the regulation of OCT4-BIRC5 or OCT4-CCND1 signaling in HCC, which may be helpful for cancer diagnosis and treatment.

## Abbreviations

AFP: Alpha-fetoprotein;DAPI: 4^′^,6-diamidino-2-phenylindole dihydrochloride;DFS: Disease-free survival;EGFP: Enhanced green fluorescent protein;HBV: Hepatitis B virus;HCC: Hepatocellular carcinoma;MOI: Multiplicities of infection;MTT: Methyl Thiazolyl Tetrazolium;OCT4: Octamer-binding transcription factor 4;OS: Overall survival;TUNEL: Terminal deoxynucleotidyl transferase-mediated dUTP nick end labeling

## Competing interests

The authors declare no conflict of interest.

## Authors’ contributions

Conceived and designed the experiments: CS; Performed the experiments: LC, CL, YY, DW, LF, HQ, JW and ZL; Contributed reagents/materials/analysis tools and analyzed the data: LC, CL, XJ, MW, JZ and CS; Wrote the paper: LC, CL, LF, JW and CS. All authors read and approved the final manuscript.

## Pre-publication history

The pre-publication history for this paper can be accessed here:

http://www.biomedcentral.com/1471-2407/13/82/prepub

## Supplementary Material

Additional file 1 Materials and methods. Click here for file

Additional file 2: Table S1 OCT4 and BIRC5 expression related to HCC clinicopathological features. Click here for file

Additional file 3: Figure S1 OCT4 and BIRC5 overexpression in clinical hepatocellular carcinoma (HCC) specimens. (A) Forty-nine pairs of cancer tissues and liver tissues were taken from clinical HCC patients. All patients underwent hepatectomy at the Eastern Hepatobiliary Surgery Hospital (Shanghai, China) between September 25, 2007, and September 30, 2009. The study patients were confirmed to have no distant metastases, detectable ascites, or chemotherapy and radiotherapy before surgery. The resected lesions were diagnosed pathologically as HCC after surgery. Paraffin-embedded consecutive sections were subjected to immunohistochemical examination for BIRC5 and OCT4 expression using the UltraSensitive Streptavidin Proxidase Kit (Fuzhou Maixin Biotechnology Development Co., Fuzhou, China), original magnification 200×. (B) OCT4 and BIRC5 expression levels were scored according to the percentages of positive cells counted within 5 high-power fields, positive cell percentages from <10% to <100% were defined as score 1 to 9, all cells that were negative were scored as zero. Patient with 2 or more than score 2 was qualified as positive case. The clinical study was performed after obtaining the informed consent from every patient and did not show the patients’ names. Click here for file

Additional file 4: Figure S2 Correlation of OCT4 and BIRC5 to prognosis of HCC patients. (A) Follow-up for HCC patients started on the operation date and ended on August 1, 2010. The median duration of follow-up was 12.67 months (ranging from 1.6 to 34.2 months). During the follow-up period, patients were monitored for tumor recurrence by examination of serum AFP, abdominal ultrasound, chest radiography, computed tomography and/or magnetic resonance imaging; finally patients were also subjected to imaging diagnosis to find recurrent tumors. The disease-free survival (DFS) was defined as the time period from the follow-up start date to the date of tumor recurrence (for the patients with tumor recurrence) or the follow-up end date (for the patients without tumor recurrence), and the overall survival (OS) was defined as the time period from the start date of follow-up to the date of death or the end date of follow-up. Survival curves were calculated by the Kaplan-Meier method, and the significant difference among the three groups was compared using log-rank test. (B) Every two groups were compared and shown in Kaplan-Meier curves. Click here for file

## References

[B1] Yamamoto H Ngan CY Monden M Cancer cells survive with survivin Cancer Sci 2008 99 1709 1714 10.1111/j.1349-7006.2008.00870.x 18537980 PMC11159325

[B2] Duffy MJ O’Donovan N Brennan DJ Gallagher WM Ryan BM Survivin: a promising tumor biomarker Cancer Lett 2007 249 49 60 10.1016/j.canlet.2006.12.020 17275177

[B3] Hu H Li Z Chen J Wang D Ma J Wang W Li J Wu H Li L Wu M Qian Q Chen J Su C P16 reactivation induces anoikis and exhibits antitumour potency by downregulating Akt/survivin signalling in hepatocellular carcinoma cells Gut 2011 60 710 721 10.1136/gut.2010.220020 20971978

[B4] Guo Y Mantel C Hromas RA Broxmeyer HE Oct-4 is critical for survival/antiapoptosis of murine embryonic stem cells subjected to stress: effects associated with Stat3/survivin Stem Cells 2008 26 30 34 10.1634/stemcells.2007-0401 17932422 PMC2818886

[B5] Okita K Ichisaka T Yamanaka S Generation of germline-competent induced pluripotent stem cells Nature 2007 448 313 317 10.1038/nature05934 17554338

[B6] Bhartiya D Kasiviswanathan S Unni SK Pethe P Dhabalia JV Patwardhan S Tongaonkar HB Newer insights into premeiotic development of germ cells in adult human testis using Oct-4 as a stem cell marker J Histochem Cytochem 2010 58 1093 1106 10.1369/jhc.2010.956870 20805580 PMC2989246

[B7] Chen YC Hsu HS Chen YW Tsai TH How CK Wang CY Hung SC Chang YL Tsai ML Lee YY Ku HH Chiou SH Oct-4 expression maintained cancer stem-like properties in lung cancer-derived CD133-positive cells PLoS One 2008 3 e2637 10.1371/journal.pone.0002637 18612434 PMC2440807

[B8] Yuan F Zhou W Zou C Zhang Z Hu H Dai Z Zhang Y Expression of Oct4 in HCC and modulation to wnt/β-catenin and TGF-β signal pathways Mol Cell Biochem 2010 343 155 162 10.1007/s11010-010-0509-3 20549546

[B9] Zhang XY Dong QG Huang JS Huang AM Shi CL Jin B Sha HF Feng JX Geng Q Zhou J Xu HL Han BH The expression of stem cell-related indicators as a prognostic factor in human lung adenocarcinoma J Surg Oncol 2010 102 856 862 10.1002/jso.21718 20818602

[B10] Jung M Peterson H Chavez L Kahlem P Lehrach H Vilo J Adjaye J A data integration approach to mapping OCT4 gene regulatory networks operative in embryonic stem cells and embryonal carcinoma cells PLoS One 2010 5 e10709 10.1371/journal.pone.0010709 20505756 PMC2873957

[B11] Zangrossi S Marabese M Broggini M Giordano R D’Erasmo M Montelatici E Intini D Neri A Pesce M Rebulla P Lazzari L Oct-4 expression in adult human differentiated cells challenges its role as a pure stem cell marker Stem Cells 2007 25 1675 1680 10.1634/stemcells.2006-0611 17379765

[B12] Boyer LA Lee TI Cole MF Johnstone SE Levine SS Zucker JP Guenther MG Kumar RM Murray HL Jenner RG Gifford DK Melton DA Jaenisch R Young RA Core transcriptional regulatory circuitry in human embryonic stem cells Cell 2005 122 947 956 10.1016/j.cell.2005.08.020 16153702 PMC3006442

[B13] Loh YH Wu Q Chew JL Vega VB Zhang W Chen X Bourque G George J Leong B Liu J Wong KY Sung KW Lee CW Zhao XD Chiu KP Lipovich L Kuznetsov VA Robson P Stanton LW Wei CL Ruan Y Lim B Ng HH The Oct4 and Nanog transcription network regulates pluripotency in mouse embryonic stem cells Nat Genet 2006 38 431 440 10.1038/ng1760 16518401

[B14] Matoba R Niwa H Masui S Ohtsuka S Carter MG Sharov AA Ko MS Dissecting Oct3/4-regulated gene networks in embryonic stem cells by expression profiling PLoS One 2006 1 e26 10.1371/journal.pone.0000026 17183653 PMC1762406

[B15] Kanwar JR Kamalapuram SK Kanwar RK Targeting survivin in cancer: patent review Expert Opin Ther Pat 2010 20 1723 1737 10.1517/13543776.2010.533657 21083520

[B16] Ryan BM O’Donovan N Duffy MJ Survivin: a new target for anti-cancer therapy Cancer Treat Rev 2009 35 553 562 10.1016/j.ctrv.2009.05.003 19559538

[B17] O’Connor DS Schechner JS Adida C Mesri M Rothermel AL Li F Nath AK Pober JS Altieri DC Control of apoptosis during angiogenesis by survivin expression in endothelial cells Am J Pathol 2000 156 393 398 10.1016/S0002-9440(10)64742-6 10666367 PMC1850029

[B18] Wobser M Keikavoussi P Kunzmann V Weininger M Andersen MH Becker JC Complete remission of liver metastasis of pancreatic cancer under vaccination with a HLA-A2 restricted peptide derived from the universal tumor antigen survivin Cancer Immunol Immunother 2006 55 1294 1298 10.1007/s00262-005-0102-x 16315030 PMC11031112

[B19] Giménez-Bonafé P Tortosa A Pérez-Tomás R Overcoming drug resistance by enhancing apoptosis of tumor cells Curr Cancer Drug Targets 2009 9 320 340 10.2174/156800909788166600 19442052

[B20] Andersen MH Svane IM Becker JC Straten PT The universal character of the tumor-associated antigen survivin Clin Cancer Res 2007 13 5991 5994 10.1158/1078-0432.CCR-07-0686 17947459

[B21] Mita AC Mita MM Nawrocki ST Giles FJ Survivin: key regulator of mitosis and apoptosis and novel target for cancer therapeutics Clin Cancer Res 2008 14 5000 5005 10.1158/1078-0432.CCR-08-0746 18698017

[B22] Cheung CH Cheng L Chang KY Chen HH Chang JY Investigations of survivin: the past, present and future Front Biosci 2011 16 952 961 10.2741/3728 21196211

[B23] Zhang R Ma L Zheng M Ren J Wang T Meng Y Zhao J Jia L Yao L Han H Li K Yang A Survivin knockdown by short hairpin RNA abrogates the growth of human hepatocellular carcinoma xenografts in nude mice Cancer Gene Ther 2010 17 275 288 10.1038/cgt.2009.68 19876077

[B24] Carrasco RA Stamm NB Marcusson E Sandusky G Iversen P Patel BK Antisense inhibition of survivin expression as a cancer therapeutic Mol Cancer Ther 2011 10 221 232 10.1158/1535-7163.MCT-10-0756 21216939

[B25] Yamanaka K Nakata M Kaneko N Fushiki H Kita A Nakahara T Koutoku H Sasamata M YM155, a selective survivin suppressant, inhibits tumor spread and prolongs survival in a spontaneous metastatic model of human triple negative breast cancer Int J Oncol 2011 39 569 575 21674125 10.3892/ijo.2011.1077

[B26] Kanwar RK Cheung CH Chang JY Kanwar JR Recent advances in anti-survivin treatments for cancer Curr Med Chem 2010 17 1509 1515 10.2174/092986710790979935 20166933

[B27] Trabulo S Cardoso AM Santos-Ferreira T Cardoso AL Simoes S Pedroso de Lima MC Survivin silencing as a promising strategy to enhance the sensitivity of cancer cells to chemotherapeutic agents Mol Pharm 2011 8 1120 1131 10.1021/mp100426e 21619051

[B28] Gritsko T Williams A Turkson J Kaneko S Bowman T Huang M Nam S Eweis I Diaz N Sullivan D Yoder S Enkemann S Eschrich S Lee JH Beam CA Cheng J Minton S Muro-Cacho CA Jove R Persistent activation of stat3 signaling induces survivin gene expression and confers resistance to apoptosis in human breast cancer cells Clin Cancer Res 2006 12 11 19 10.1158/1078-0432.CCR-04-1752 16397018

[B29] Kim PJ Plescia J Clevers H Fearon ER Altieri DC Survivin and molecular pathogenesis of colorectal cancer Lancet 2003 362 205 209 10.1016/S0140-6736(03)13910-4 12885482

[B30] Peng XH Karna P Cao Z Jiang BH Zhou M Yang L Cross-talk between epidermal growth factor receptor and hypoxia-inducible factor-1α signal pathways increases resistance to apoptosis by up-regulating survivin gene expression J Biol Chem 2006 281 25903 25914 10.1074/jbc.M603414200 16847054 PMC3132567

[B31] Li F Altieri DC Transcriptional analysis of human survivin gene expression Biochem J 1999 344 305 311 10.1042/0264-6021:3440305 10567210 PMC1220645

[B32] Esteve PO Chin HG Pradhan S Molecular mechanisms of transactivation and doxorubicin-mediated repression of survivin gene in cancer cells J Biol Chem 2007 282 2615 2625 17124180 10.1074/jbc.M606203200

[B33] O’Connor DS Grossman D Plescia J Li F Zhang H Villa A Tognin S Marchisio PC Altieri DC Regulation of apoptosis at cell division by p34cdc2 phosphorylation of survivin Proc Natl Acad Sci USA 2000 97 13103 13107 10.1073/pnas.240390697 11069302 PMC27185

[B34] Matthai C Horvat R Noe M Nagele F Radjabi A van Trotsenburg M Huber J Kolbus A Oct-4 expression in human endometrium Mol Hum Reprod 2006 12 7 10 10.1093/molehr/gah254 16421218

[B35] Mueller T Luetzkendorf J Nerger K Schmoll HJ Mueller LP Analysis of OCT4 expression in an extended panel of human tumor cell lines from multiple entities and in human mesenchymal stem cells Cell Mol Life Sci 2009 66 495 503 10.1007/s00018-008-8623-z 19023518 PMC11131475

[B36] Gu G Yuan J Wills M Kasper S Prostate cancer cells with stem cell characteristics reconstitute the original human tumor *in vivo* Cancer Res 2007 67 4807 4815 10.1158/0008-5472.CAN-06-4608 17510410

[B37] Atlasi Y Mowla SJ Ziaee SA Bahrami AR OCT-4, an embryonic stem cell marker, is highly expressed in bladder cancer Int J Cancer 2007 120 1598 1602 10.1002/ijc.22508 17205510

[B38] Chiou SH Yu CC Huang CY Lin SC Liu CJ Tsai TH Chou SH Chien CS Ku HH Lo JF Positive correlations of Oct-4 and Nanog in oral cancer stem-like cells and high-grade oral squamous cell carcinoma Clin Cancer Res 2008 14 4085 4095 10.1158/1078-0432.CCR-07-4404 18593985

[B39] Chang CC Shieh GS Wu P Lin CC Shiau AL Wu CL Oct-3/4 expression reflects tumor progression and regulates motility of bladder cancer cells Cancer Res 2008 68 6281 6291 10.1158/0008-5472.CAN-08-0094 18676852

[B40] Mityaev MV Kopantzev EP Buzdin AA Vinogradova TV Sverdlov ED Functional significance of a putative sp1 transcription factor binding site in the survivin gene promoter Biochemistry (Mosc) 2008 73 1183 1191 10.1134/S0006297908110035 19120021

[B41] Jiang Y Saavedra HI Holloway MP Leone G Altura RA Aberrant regulation of survivin by the RB/E2F family of proteins J Biol Chem 2004 279 40511 40520 10.1074/jbc.M404496200 15271987

[B42] Tapias A Ciudad CJ Roninson IB Noé V Regulation of Sp1 by cell cycle related proteins Cell Cycle 2008 7 2856 2867 10.4161/cc.7.18.6671 18769160 PMC2810142

