# Supplementary material for: OCT4 increases BIRC5 and CCND1 expression and promotes cancer progression in hepatocellular carcinoma
Source: BMC Cancer. 2013 Feb 22;13:82. doi: 10.1186/1471-2407-13-82 (PMC3583731; doi:10.1186/1471-2407-13-82)
Supplement: Additional file 1 — Materials and methods. [file 1471-2407-13-82-S1.doc]

**Additional materials and methods**

1. **Cell lines and cell culture**

Human HCC cell lines Hep3B, HepG2, PLC/PRF5 and SK-Hep-1 and the human normal fibroblast cell line BJ were purchased from the American Type Culture Collection (Manassas, VA, USA). Human HCC cell lines SMMC-7721, BEL-7402 and BEL-7404 were obtained from the Type Culture Collection of the Chinese Academy of Sciences (Shanghai, China). All cell lines were cultured in media recommended by the providers. All media were supplemented with 10 % heat-inactivated fetal bovine serum containing 100 units/ml penicillin and 100 µg/ml streptomycin.

1. **Amplification of adenoviruses**

The recombinant adenoviruses, Ad5-OCT4, Ad5-shBIRC5, Ad5-shOCT4 and AdDual-shRNA. were amplified in HEK293 cells, purified by ultra-centrifugation in caesium chloride (CsCl) gradients, and viral titres were measured using the tissue culture infectious dose 50 (TCID_50_) method.

1. **Determination of gene expression in cultured cells**

Cells were cultured in 6-well plates at a density of 10^5^ cells/well and infected with adenoviruses (Ad5-OCT4) at multiplicities of infection (MOI) of 20 pfu/cell and harvested 48 h after infection. [Simultaneously](app:ds:simultaneously), cells were transfected with shRNA vectors at a concentration of 20 µg/well using the Lipofectamine 2000 reagent (Invitrogen Corporation Shanghai Representative Office, Shanghai, China). After 24 h of transfection, G418 (400 μg/ml) was added to select for shRNA transfectants. After continuous culturing for 24 h, cells were harvested. The parental and transfectant cells were examined in cell lysates by Western blotting using the rabbit anti-BIRC5 (R&D Systems Inc., Miles, CA), mouse anti-OCT4 (Santa Cruz Biotechnology, Inc., Santa Cruz, CA) and mouse anti-CCND1 (Cell Signaling Technology, Inc., Beverly, MA) primary antibodies.

The parental, virus-infected and shRNA-transfected cells were cultured in Lab-Tek chamber slides (Electron Microscopy Sciences, Hatfield, PA) at a density of 10^4^ cells/well for 48 h and then fixed in 4 % formaldehyde for 30 min. After washing with PBS (0.1 M, pH 7.2), the slides were incubated with the primary antibodies (anti-OCT4 and anti-BIRC5) at 4 °C overnight and then with secondary antibodies (FITC-conjugated anti-rabbit IgG and TRITC-conjugated anti-mouse IgG; Santa Cruz Biotechnology, Inc., Santa Cruz, CA) at room temperature for 1 h. 4’,6-diamidino-2-phenylindole dihydrochloride (DAPI) was used to stain cellular nuclei. Cells were observed under a fluorescence microscope.

1. **Detection of promoter activity in cell lines**

The parental, virus-infected and shRNA-transfected cells were seeded on 24-well plates at a density of 5×10^4^ cells/well, cultured overnight, and then transfected with the luciferase expression plasmids (pSRVN-Luc,^2^ pGL3wPro-Luc, pGL3mPro-Luc and pGL3ePro-Luc) at 200 ng/well using the Lipofectamine 2000 reagent (Invitrogen Corporation Shanghai Representative Office). The control plasmid pRL-TK (20 ng/well), which contained the *Renilla* luciferase gene under the control of the herpes simplex virus thymidine kinase promoter, was co-transfected with the luciferase expression plasmids to standardize the transcription efficiency. Luciferase assays were performed using the Dual-Luciferase Reporter Assay System (Promega Corp., Madison, WI) on a MiniLumat LB-9506 Luminometer (Berthold Technologies, Bad Wildbad, Germany).

Cells were seeded on 96-well plates at a density of 5×10^3^ cells/well, cultured overnight, and then transfected with the EGFP expression plasmid pSRVN-EGFP at 2 μg/well using the Lipofectamine 2000 reagent (Invitrogen Corporation Shanghai Representative Office). After 48 h of transfection, cells were observed under a ﬂuorescence microscope.

1. **Measurement of cell viability by methyl thiazolyl tetrazolium (MTT) assay**

The parental and shRNA-transfected Hep3B cells were cultured for 48 h or 72 h after transfection in 96-well plates at a density of 5×10^3^ cells/well. Cell viability was then measured using 3-(4,5-dimethylthiazolyl-2)-2,5-diphenyltetrazolium bromide (MTT Regent M2128, Sigma-Aldrich Shanghai Trading Co., Shanghai, China) according to the [manufacturer](app:lj:生产商?ljtype=blng&ljblngcont=0&ljtran=manufacturer)’s protocol. The average absorbance for each sample was examined with a microplate reader (SmartSpec 3000, BIO-RAD Laboratories, Hercules, CA) at a wavelength of 570 nm with a reference wavelength of 655 nm.

1. **Flow cytometric analysis of cultured cells**

The parental, virus-infected and shRNA-transfected cells were cultured and harvested. Cells were resuspended at 10^6^ cells/ml and stained with a phycoerythrin (PE)-labeled mouse anti-CD133 antibody (Milteny Biotec, Bergisch Gladbach, Germany) to detect CD133 expression stained with propidium iodide (PI) alone to detect the cell cycle, or were the cells stained with PI and Annexin V-FITC to detect cell apoptosis using the Annexin V-FITC/PI Apoptosis Detection Kit (Alpha Diagnositic International, San Antonio, TX) according to the kit protocol. All prepared cells were quantified with a flow cytometer (FACSAria, BD Biosciences, San Diego, CA).

1. **Pathological examinations of xenograft tumors**

On day 39 after the treatments, the [maximal](app:ds:maximal) xenograft tumors exceeded the weight approved by the Animal Care Committee, and therefore, the mice were killed, and the tumours were removed and weighed. Tumor specimens were fixed in 4 % paraformaldehyde for 4 h, and paraffin-embedded consecutive sections were cut for detecting OCT4 and BIRC5 expression by immunohistochemistry and for counting the apoptotic cells by TUNEL labeling (In Situ Cell Death Detection Kit, Fuzhou Maixin Biotechnology Development Co., Fuzhou, China); Epon812-embedded ultrathin sections were prepared for observing apoptotic cell ultrastructures using a transmission electron microscope.
