# Supplementary material for: OCT4 increases BIRC5 and CCND1 expression and promotes cancer progression in hepatocellular carcinoma
Source: BMC Cancer. 2013 Feb 22;13:82. doi: 10.1186/1471-2407-13-82 (PMC3583731; doi:10.1186/1471-2407-13-82)
Supplement: Additional file 2: Table S1 — OCT4 and BIRC5 expression related to HCC clinicopathological features. [file 1471-2407-13-82-S2.doc]

**Additional Table S1**

**Table S1 OCT4 and BIRC5 expression related to HCC clinicopathological features**

| **Parameters** | **n** | | **OCT4+/BIRC5+**  **(n=12)** | **OCT4-/BIRC5+**  **(n=26)** | **OCT4-/BIRC5-**  **(n=11)** |
| --- | --- | --- | --- | --- | --- |
| Gender |  |  |  |  |  |
| Male |  | 37 | 9 | 21 | 7 |
| Female |  | 12 | 3 | 5 | 4 |
| Age ^a^ |  |  |  |  |  |
| <49 (y) |  | 24 | 6 | 12 | 6 |
| ≥49 (y) |  | 25 | 6 | 14 | 5 |
| HBV ^b^ |  |  |  |  |  |
| Positive |  | 45 | 11 | 23 | 11 |
| Negative |  | 4 | 1 | 3 | 0 |
| AFP |  |  |  |  |  |
| ≥40 μg/L |  | 35 | 10 | 16 | 9 |
| <40 μg/L |  | 14 | 2 | 10 | 2 |
| Cancer Thrombi ^c^ |  |  |  |  |  |
| Positive |  | 21 | 9 | 9 | 3 |
| Negative |  | 28 | 3 | 17 | 8 |
| Tumor Size ^d^ |  |  |  |  |  |
| <5.2 cm |  | 17 | 4 | 9 | 4 |
| ≥5.2 cm |  | 32 | 8 | 17 | 7 |
| Cell Differentiation |  |  |  |  |  |
| Grade II |  | 12 | 3 | 4 | 6 |
| Grade III |  | 37 | 9 | 22 | 5 |
| DFS ^d^ |  |  |  |  |  |
| <6.5 (m) |  | 30 | 8 | 20 | 2 |
| ≥6.5 (m) |  | 19 | 4 | 6 | 9 |
| OS ^d^ |  |  |  |  |  |
| <16.2 (m) |  | 27 | 8 | 16 | 2 |
| ≥16.2 (m) |  | 22 | 4 | 10 | 9 |

**Notes:** Grouping according to ^a^[median](app:lj:中位数?ljtype=blng&ljblngcont=0&ljtran=median) value (y: years), ^b^HBsAg positive or negative, ^c^portal vein and bile duct thrombi, ^d^mean value (m: months)*.*
