# Supplementary figures and images for: OCT4 increases BIRC5 and CCND1 expression and promotes cancer progression in hepatocellular carcinoma
Source: BMC Cancer. 2013 Feb 22;13:82. doi: 10.1186/1471-2407-13-82 (PMC3583731; doi:10.1186/1471-2407-13-82)

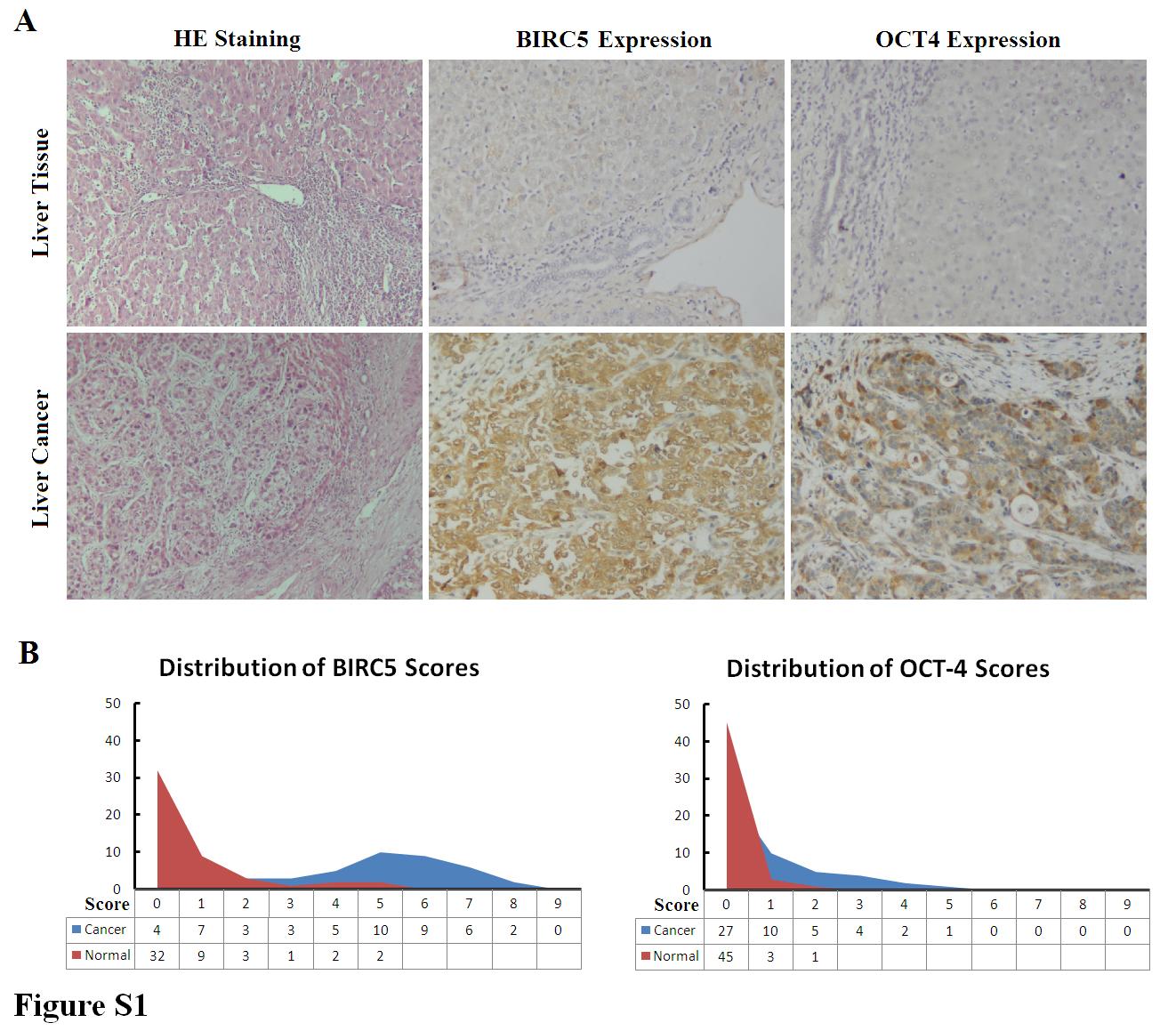

Supplement: Additional file 3: Figure S1 — OCT4 and BIRC5 overexpression in clinical hepatocellular carcinoma (HCC) specimens. (A) Forty-nine pairs of cancer tissues and liver tissues were taken from clinical HCC patients. All patients underwent hepatectomy at the Eastern Hepatobiliary Surgery Hospital (Shanghai, China) between September 25, 2007, and September 30, 2009. The study patients were confirmed to have no distant metastases, detectable ascites, or chemotherapy and radiotherapy before surgery. The resected lesions were diagnosed pathologically as HCC after surgery. Paraffin-embedded consecutive sections were subjected to immunohistochemical examination for BIRC5 and OCT4 expression using the UltraSensitive Streptavidin Proxidase Kit (Fuzhou Maixin Biotechnology Development Co., Fuzhou, China), original magnification 200×. (B) OCT4 and BIRC5 expression levels were scored according to the percentages of positive cells counted within 5 high-power fields, positive cell percentages from <10% to <100% were defined as score 1 to 9, all cells that were negative were scored as zero. Patient with 2 or more than score 2 was qualified as positive case. The clinical study was performed after obtaining the informed consent from every patient and did not show the patients’ names. [file 1471-2407-13-82-S3.jpeg]

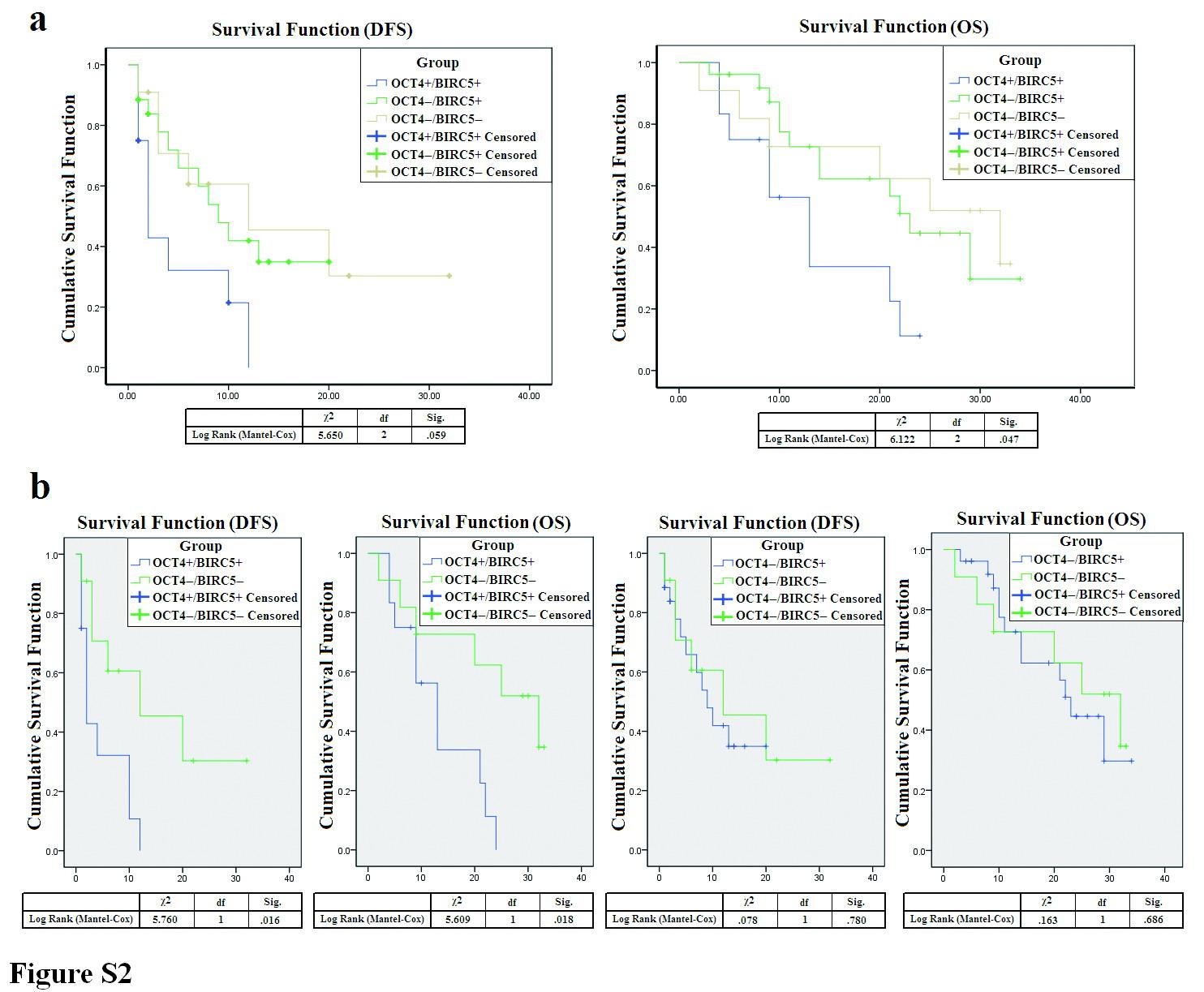

Supplement: Additional file 4: Figure S2 — Correlation of OCT4 and BIRC5 to prognosis of HCC patients. (A) Follow-up for HCC patients started on the operation date and ended on August 1, 2010. The median duration of follow-up was 12.67 months (ranging from 1.6 to 34.2 months). During the follow-up period, patients were monitored for tumor recurrence by examination of serum AFP, abdominal ultrasound, chest radiography, computed tomography and/or magnetic resonance imaging; finally patients were also subjected to imaging diagnosis to find recurrent tumors. The disease-free survival (DFS) was defined as the time period from the follow-up start date to the date of tumor recurrence (for the patients with tumor recurrence) or the follow-up end date (for the patients without tumor recurrence), and the overall survival (OS) was defined as the time period from the start date of follow-up to the date of death or the end date of follow-up. Survival curves were calculated by the Kaplan-Meier method, and the significant difference among the three groups was compared using log-rank test. (B) Every two groups were compared and shown in Kaplan-Meier curves. [file 1471-2407-13-82-S4.jpeg]
